# Supplementary material for: Male partner involvement in postnatal care service utilization and associated factors: A community-based cross-sectional study in Motta district, Northwest Ethiopia
Source: PLoS One. 2023 Jan 20;18(1):e0276477. doi: 10.1371/journal.pone.0276477 (PMC9858086; doi:10.1371/journal.pone.0276477)
Supplement: S1 Appendix — (DOCX) [file pone.0276477.s001.docx]

**Appendix: Questionnaires**

**English Version**

**Part I: Socio-demographic characteristics of the respondent**

| No | Question and Filters | Coding Categories | Skip to |
| --- | --- | --- | --- |
| 101 | How old are you now? | ______ years |  |
| 102 | What religion do you practice? | 1. Orthodox  2. Muslim  3. Protestant  4. Catholic  5. Others(specify)_______ |  |
| 103 | What is your Residence? | 1. Urban 2. Rural |  |
| 104 | What is your Ethnicity? | 1. Amhara  2. Tigre  3. Oromo  4. Others(specify)_______ |  |
| 105 | What is your educational status? | 1. No formal education 2. Primary (1-8) 3. Secondary(9-12) 4. College and above |  |
| 106 | What is the educational status of your wife? | 1. No formal education   1. Primary (1-8) 2. Secondary(9-12) 3. College and above |  |
| 107 | What is your occupational status? | 1. Farmer 2. Student 3. Merchant 4. Governmental worker 5. Daily laborer 6. If other(specify)_______ |  |
| 108 | What is the occupational status of your wife? | 1. Housewife 2. Farmer 3. Student 4. Merchant 5. Governmental worker 6. Daily laborer   6. If other(specify)_______ |  |
| 109 | How many children do you have? | ______ |  |

**Part II: Wealth index**

1. **Wealth index/ income questions for urban residence**

| 201 | Owner of the house | 1. Private 3. Rents  2. kebele’s 4. Other______ |
| --- | --- | --- |
| 202 | House floor | 1. soil/teter 3. cement  2. Muck 4.ceramics |
| 203 | House roof | 1. Corrugated iron 2. Grass 3. Other … |
| 204 | Houses outside the wall | 1. Stone with mud 2. Wood with mud 3. stone/blocket with cement  4. Others ______ |
| 205 | Berth | In Number______ |
| 206 | Which of the following types of fuel does your household use mainly? | 1. Electricity 4. Animal Dug  2. Charcoal 5. Gas  3. Wood 6. Others______ |
| 207 | What types of toilet do your household use? | 1. Flush or pour-flush latrine dwelling  2. Ventilated improved pit latrine 3. Traditional latrine 4. Public latrine 5. Space field |
| 208 | Kitchen house | 1.Yes 2.No |
| 209 | Source of water | 1. Piped water into the compound  2. Public Borehole  3. Public tap  4.Borehole with hand pump into the compound  5. Dam from rain source  6. pond/river/race/head  7. Borehole without hand pump into the compound  8. Dam from the river source  9. Others______ |
| 210 | Do any of your household members have the following? If yes how many? | |
|  | Radio | 1. Yes 2. No |
|  | Television | 1. Yes 2. No |
|  | non-mobile telephone | 1. Yes 2. No |
|  | Refrigerator | 1. Yes 2. No |
|  | Bed /cotton/sponge/spring matters | 1. Yes 2. No |
|  | Table | 1. Yes 2. No |
|  | Chair | 1. Yes 2. No |
| 211 | Does any of your household members have the following? | |
|  | Mobile Phone | 1. Yes 2. No |
|  | Motor Cycle | 1. Yes 2. No |
|  | Bajaj | 1. Yes 2. No |
|  | Animal draw cart | 1. Yes 2. No |
|  | Car or truck | 1. Yes 2. No |
|  | oxen/cow | 1. Yes 2. No |
|  | Horse/mule | 1. Yes 2. No |
|  | Goat/Sheep | 1. Yes 2. No |
|  | Hen | 1. Yes 2. No |
|  | Beehives | 1. Yes 2. No |
|  | Bank account/ Saving Book | 1. Yes 2. No |

1. **Wealth index question for rural residence**

| 212 | House roof | 1. Corrugated iron 2. Grass/wood 3. Others ______ |
| --- | --- | --- |
| 213 | Main source of houses’ cooking | 1. Electricity 3. Wood 2. Charcoal 4. Animal Dug  5. Gas 6. Others______ |
| 21 | Does the household own separate house for domestic animals | 1. Yes 2. No |
| 216 | Kitchen house | 1 Yes 2. No |
| 217 | Source of water | 1. Piped water into the compound  2. public Borehole  3 Public tap  4. Borehole with hand pump into the compound  5. Dam from rain source  6.pond/river/race/headwaters  7. Borehole without hand pump into the compound  8. Dam from the river source  9. Others ______ |
| 218 | Does any member of your household have the following? If yes how many? |  |
|  | Radio or Tape | 1. Yes 2. No |
|  | Bed/ cotton/ sponge/spring/ mattress | 1. Yes 2. No |
|  | Mobile Phone | 1. Yes 2. No |
|  | Water generator | 1. Yes 2. No |
|  | Solar energy | 1. Yes 2. No |
|  | Other(specify) _________ |  |
| 219 | Domesticated animals | |
|  | Ox/cow | 1. Yes 2.No |
|  | Calf | 1. Yes 2.No |
|  | Donkey | 1. Yes 2.No |
|  | horse/mule | 1. Yes 2.No |
|  | Goat/sheep | 1. Yes 2.No |
|  | Hen | 1. Yes 2.No |
|  | Beehives | 1. Yes 2.No |
| 220 | Does the following agricultural products produced in the last year (2019)? If yes how much in quintal? | |
|  | Teff | 1. Yes 2.No |
|  | Barley | 1. Yes 2.No |
|  | Wheat | 1. Yes 2.No |
|  | Maize | 1. Yes 2.No |
|  | Millet | 1. Yes 2.No |
|  | Sesame Seed | 1. Yes 2.No |
|  | Bean | 1. Yes 2.No |
|  | Pea | 1. Yes 2.No |
|  | Chickpea | 1. Yes 2.No |
|  | Lentil | 1. Yes 2.No |
|  | Dagusa | 1. Yes 2.No |
| 221 | If there are other productions (list) |  |

**Part III: Males partners’ knowledge about PNC service**

| No | Question and Filters | Coding Categories | Skip to |
| --- | --- | --- | --- |
| 301 | What is the recommended number of PNC visit does the mother have after giving birth? | 1.one 2.two  3.three  4.four  5.more than four  6. don’t know |  |
| 302 | When these PNC visits provided? | ----------------- |  |
| 303 | What do you know about PNC services? | 1. Check up 2. Family planning 3. Immunization 4. Early detection of complication and prompt of treatment 5. Counseling |  |
| 304 | What is your source of information? | 1.Family members  2.HEW  3.Health professionals  4.Other sources ________ |  |
| 305 | Do you think that visiting health  institutions during the Postnatal period is important for the mother and child? | 1. Yes 2. No |  |
| 306 | Do you think that PNC is important even if there is no complication? | 1. Yes 2. No |  |

**Part IV: Male partner’s knowledge about danger sign during the postnatal period**

| No | Question and Filters | Coding Categories | Skip to |
| --- | --- | --- | --- |
| 401 | What danger signs could be occurred during postpartum period for the mother? | 1. Fever 2.Heavy headache  3.Heavy vaginal bleeding 4.Foul smelling vaginal discharge 5.Blurring of vision  6.Depresion  7. Breast and calf redness and pain |  |
| 402 | What danger signs could be occurred during postpartum period for the mother? | 1. Fever  2. Feels cold  3.Difficulty of breathing 4.Abnormal body movement 5.Cord with pus and blood 6.Difficulty of feeding  7.yellow palms/soles/eyes |  |
| 403 | What are the complications that occur during the PNC period? | 1.pregnancy induced hypertension  2.Breast and uterine infection  3.Postpartum bleeding  4.DVT  5.Postpartal psychosis  6. Wound site infection |  |

**Part V: Male partner’s attitude towards PNC service utilization**

| No | Statements | Strongly agree | Agree | Neutral | Disagree | Strongly Disagree |
| --- | --- | --- | --- | --- | --- | --- |
| 501 | It is the waste of time for male partners to participate with their wives during PNC visit |  |  |  |  |  |
| 502 | Male partners should give emotional, financial, and physical support for their wives during PNC period |  |  |  |  |  |
| 503 | Male partners must focus on job responsibilities during the postnatal period rather than to involve in caring for their wives and child |  |  |  |  |  |
| 504 | PNC services should only be left to women alone |  |  |  |  |  |
| 505 | Visiting health institutions during the postnatal period is important for mothers and their children. |  |  |  |  |  |
| 506 | Recommend other males to involve in PNC service |  |  |  |  |  |
| 507 | Mothers should follow PNC follow up within 42 days |  |  |  |  |  |

**Part VI: Cultural Barriers of male partner involvement in PNC service utilization**

| No | Question and Filters | Coding Categories | Skip to |
| --- | --- | --- | --- |
| 601 | Postnatal care is the domain of women/ women’s affairs? | 1.Yes 2.No  3.Don’t know |  |
| 602 | PNC clinic is a place where the service is given only for women | 1.Yes 2.No  3.Don’t know |  |
| 603 | Is there cultural communication/discussion in maternal health service utilization in your family? | 1. Yes 2.No |  |
| 604 | Is there any misconception or myth about male involvement in maternal health service utilization | 1. Yes 2.No |  |

**Part VII: Health service related characteristics**

| No | Question and Filters | Coding Categories | Skip to |
| --- | --- | --- | --- |
| 701 | How far is the health facility that gives PNC services in time?(in time) | 1.≤ 30 minutes  2.>30 minutes |  |
| 702 | Do you get transportation services any time to reach to a health facility for PNC visit | 1.Yes 2.No  3.Don’t know |  |
| 703 | Had health professionals have good welcoming for males in maternal health service utilization | 1.Yes 2.No  3.Don’t know |  |
| 704 | Is health care provider not allowed to the room other than health workers to ensure privacy? | 1.Yes 2.No  3.Don’t know |  |
| 705 | How much time you stay at the health facility/clinic to get PNC services ? | 1. ≤ 1hr 2. > 1hr  3. Don’t know |  |

**Part VIII: Magnitude of male partner involvement in PNC service utilization**

| No | Question and Filters | Coding Categories | Skip to |
| --- | --- | --- | --- |
| 801 | Have you discussed the importance of PNC service with your partner? | 1.Yes 2.No |  |
| 802 | Did you have shared decision-making powers on PNC with your partner | 1.Yes 2.No |  |
| 803 | Did you accompany your partner for the PNC service? | 1.Yes 2.No |  |
| 804 | If say no, What is your reason? | 1.being preoccupied with work  2. believe that PNC is a concern of the wife  3. Do not know PNC could result in a complication  4. do not feel responsible  5.feel shame  6. Women do not allow their partner to accompany  7. Others, specify----------- |  |
| 805 | Did you discuss on postpartum contraception with your partner? | 1.Yes 2.No |  |
| 806 | Did you have a discussion with the health care provider about PNC services complications during the postnatal period? | 1.Yes 2.No |  |
| 807 | Have you provided physical support for your for PNC service utilization? | 1.Yes 2.No |  |
| 808 | Have you provided emotional support to your partner (encourage) for PNC service utilization? | 1.Yes 2.No |  |
| 809 | Have you provided financial support to your partner for PNC service utilization? | 1.Yes 2.No |  |
| 810 | What is your role during the PNC period? | 1. Took care of domestic chores household tasks  2.Looked after the children at home |  |

**ክፍል አንድ፡ ስነ-ህዝብና ማሀበራዊ ጉዳዮችን የሚመለከቱ ጥያቄዎች**

| ተ.ቁ | ጥያቄዎች | አማራጭ መልሶች | ይለፉ |
| --- | --- | --- | --- |
| 101 | እድሜ? | ______ዓመት |  |
| 102 | የሚኖሩበት ቦታ? | 1.ገጠር 2. ከተማ |  |
| 103 | ሀይማኖት? | 1.ኦርቶዶክስ ክርስቲያን  2. ሙስሊም  3. ካቶሊክ  4.ፕሮቴስታንት  5. ሌላ ካለ ይጠቀስ______ |  |
| 104 | ብሔር? | 1.አማራ 2. ኦሮሞ 3. ትግሬ 4. ሌላ ካለ______ |  |
| 105 | የትምህርት ደረጃ? | 1.መደበኛ ትምህርት ያልተማረ  2. 1ኛ ደረጃ(1-8ኛ)  3.2ኛ ደረጃ(9-12ኛ)  4. ኮሌጅና ከዚያ በላይ |  |
| 106 | ስራ? | 1.ገበሬ  2. ተማሪ  3. የግል ስራ/ነጋዴ  4. ተቀጣሪ(የመንግስት/የግል)  5. የቀን ሰራተኛ  6. ሌላ ካለ |  |
| 107 | የባለቤትዎ የትምህርት ደረጃ? | 1.መደበኛ ትምህርት ያልተማረ  2. 1ኛ ደረጃ(1-8ኛ)  3.2ኛ ደረጃ(9-12ኛ)  4. ኮሌጅና ከዚያ በላይ |  |
| 108 | የባለቤትዎ ስራ? | 1. የቤት እመቤት  2. ገበሬ  3. ተማሪ  4. የግል ስራ/ነጋዴ  5. ተቀጣሪ(የመንግስት/የግል)  6. የቀን ሰራተኛ  7. ሌላ ካለ |  |
| 109 | ስንት ልጆች አላችሁ? | ______ |  |

**ክፍል 2፡ የቤተሰብ የሀብት ሁኔታን የሚመለከት ጥያቄ**

**ሀ. ለከተማ ነዋሪወች ብቻ**

| 201 | የቤቱ ይዞታ የማን ነዉ? | 1. የግል 2.የቀበሌ 3. የክራይ 4.ሌላ______ |
| --- | --- | --- |
| 202 | የቤቱ ወለል | 1. አፈር/ጠጠር 2. በዕበት የተለቀለቀ 3.ሲሚንቶ 4. ሴራሚክ |
| 203 | የቤቱ ጣራ | 1. ቆርቆሮ 2. ሳር 3. ሌላ ….. |
| 204 | የቤቱ የውጭ ግድግዳ | 1. ድንጋይ በጭቃ 2. ድንጋይ/ብሎኬት በስሚነቶ  3. ዕንጨት በጭቃ 4. ሌላ ______ |
| 205 | ለመኝታ የሚያገለግል ክፍል ብዛት | በቁጥር ______ |
| 206 | የቤቱ ዋና የምግብ ማብሰያ | 1. ኤሌክትሪክ 2. እንጨት 3. ጋዝ  4. ከሰል 5. ኩበት 6. ሌላ____ |
| 207 | የሚጠቀሙት መጸዳጃ ቤት ምን ዐይነት ነው | 1. የውሃ መልቀቂያና ማፋሰሻ ያለው የግል  2. ባህላዊ የጉድገጓድ 3.የተሻሻል ሽታ አልባ የጉድጓ  4. የጋራ 5. ሜዳ ላይ |
| 208 | የምግብ ማብሰያ ቤት አልዎት | 1.አወ 2.የለም |
| 209 | የሚጠቀሙበት የውኃ ምንጭ ምንድን ነው | 1. በግቢው ውስጥ ካለ ቧንቧ  2. የተጠራቀመ የዝናብ ውኃ 3. የጋራ ጉድጓድ  4.ኩሬ/ወንዝ/ወራጅ/ምንጭ 5.የጋራ ቧንቧ  6. የጉድጓድ ውኃ ፓምፕ የሌለው  7.ግቢዎ ውስጥ የጉድጓድ ውኃ ከነፓንፑ |
| 210 | ቤት ውስጥ ከዚህ በታች የተዘረዘሩት አሉ? አወ ካሉ ቁጥራቸው ምን ያህል ነው |  |
|  | ሬዲዮ | 1. አወ 2. የለም |
|  | ቴለተቪዥን | 1. አወ 2. የለም |
|  | የቤት ስልክ | 1. አወ 2. የለም |
|  | ፍሪጅ | 1. አወ 2. የለም |
|  | አልጋ /የጥጥ/ ስፖንጅ/ስፕሪንግፍራሽ | 1. አወ 2. የለም |
|  | ጠረጴዛ | 1. አወ 2. የለም |
|  | ወንበር | 1. አወ 2. የለም |
| 211 | ከቤተሰብዎ ውስጥ ከሚከተሉት ያለው አለ? | |
|  | የዕጅ ስልክ | 1. አወ 2. የለም |
|  | ሞተር ሳይክል | 1. አወ 2. የለም |
|  | ባጃጅ | 1. አወ 2. የለም |
|  | ጋሪ | 1. አወ 2. የለም |
|  | መኪና | 1. አወ 2. የለም |
|  | በሬ/ላም | 1. አወ 2. የለም |
|  | ፈረስ/በቅሎ | 1. አወ 2. የለም |
|  | ፍየል/በግ | 1. አወ 2. የለም |
|  | ደሮ | 1. አወ 2. የለም |
|  | የንብ ቀፎ | 1. አወ 2. የለም |
|  | የባንክ/የቁጠባ ደብተር | 1. አወ 2. የለም |

**ለ. ለገጠር ነዋሪወች የሚሞላ**

| 212 | የቤቱ ጣራ | | 1. ቆርቆሮ 2. ሳር/ዕንጨት 3. ሌላ ____ |
| --- | --- | --- | --- |
| 213 | የቤቱ ዋና የምግብ ማብሰያ | | 1. ኤሌክትሪክ 2. ከሰል 3. እንጨት 4. ኩበት 5. ጋዝ 6. ሌላ ____ |
| 214 | ለእንስሳቱ መኖሪያ የሚሆን የተለየ ቤት አልዎት | | 1. አወ 2. የለም |
| 215 | የምግብ ማብሰያ ቤት አልዎት | | 1.አዎ 2.የለም |
| 217 | የሚጠቀሙበት የውኃ ምንጭ ምንድን ነው | | 1. በግቢው ውስጥ ካለ ቧንቧ  2.ኩሬ/ወንዝ/ወራጅ/ምንጭ  3. የጋራ ጉድጓድ4. የጉድጓድ ውኃ ፓምፕ የሌለው  5.የጋራ ቧንቧ  6.የተገደበ ውሃ  7.ግቢዎ ውስጥ የጉድጓድ ውኃ ከነፓንፑ  8. የተጠራቀመ የዝናብ ውኃ  9. ሌላ ______ |
| 218 | ቤት ውስጥ ከዚህ በታች የተዘረዘሩት አሉ? | | |
|  | ሬዲዮ ወይም ቴፕ | 1. አወ 2.የለም | |
|  | አልጋ/የጥጥ/ ስፖንጅ/ስፕሪንግፍራሽ | 1. አወ 2.የለም | |
|  | የሞባይል ስልክ | 1. አወ 2.የለም | |
|  | የውኃ ጄኔሬተር | 1. አወ 2.የለም | |
|  | የሶላር ሀይል | 1. አወ 2.የለም | |
|  | ሌላ ካለ ይጥቀሱ |  | |
| 219 | የቤት እንሰሳ |  | |
|  | በሬ/ላም | 1. አወ………. 2.የለም | |
|  | ጥጃ | 1. አወ………. 2.የለም | |
|  | አህያ | 1. አወ………. 2.የለም | |
|  | ፈረስ/በቅሎ | 1. አወ………. 2.የለም | |
|  | ፍየል/በግ | 1. አወ………. 2.የለም | |
|  | ደሮ | 1. አወ………. 2.የለም | |
|  | የንብ ቀፎ | 1. አወ………. 2.የለም | |
| 220 | ከዚህ በታች የተጠቀሱት የእርሻና የጓሮ ምርት ባለፈው አመት(2012) አምርተዋል አወ ካሉ በኩንታል ምን ያህል አመረቱ | | |
|  | ጤፍ | 1. አወ………. 2.የለም | |
|  | ገብስ | 1. አወ………. 2.የለም | |
|  | ስንዴ | 1. አወ………. 2.የለም | |
|  | በቆሎ | 1. አወ………. 2.የለም | |
|  | ማሽላ | 1. አወ………. 2.የለም | |
|  | ሰሊጥ | 1. አወ………. 2.የለም | |
|  | ባቄላ | 1. አወ………. 2.የለም | |
|  | አተር | 1. አወ………. 2.የለም | |
|  | ሽንብራ | 1. አወ………. 2.የለም | |
|  | ምስር | 1. አወ………. 2.የለም | |
|  | ዳጉሳ | 1. አወ………. 2.የለም | |
|  | ሌሎች ምርቶች ካሉ የጥቀሱ |  | |

**ክፍል ሶስት፡ የወንድ አጋር ስለደህረ-ወሊድ ክትትል ያላቸውን እውቀት የሚመለከት ጥያቄ**

| 301 | ለእናቶች የሚመከረው የድህረ-ወሊድ ክትትል ስንት ነው ብለው ያስባሉ? | 1.አንድ  2.ሁለት  3.ሶስት  4.አራት  5.ከአራት በላይ  6.አላቅም |  |
| --- | --- | --- | --- |
| 302 | ክትትል የሚያደረጉት መቸ መቸ ነው? | ……….. |  |
| 303 | በድህረ-ወሊድ ክትትል ጊዜ ስለሚሰጡ አገልግሎቶች ምን ምን ያቃሉ? | 1. የምርመራ አገልግሎት  2. የወሊድ መቆጣጠያ አገልግሎት 3. የክትባት አገልግሎት  4. በድህረ-ወሊድ ጊዜ የሚከሰቱ ችግሮችን ቶሎ ማከም  5. የምክር አገልግሎት |  |
| 304 | መረጃውን ከየት አገኙ? | 1. ከቤተሰብ  2. ከጤና ባለሙያ  3. ከጎረቤት/ከዘመድ/ከጋደኛ  4. ከሌሎች ምንጮች……. |  |
| 305 | የድህረ-ወሊድ ክትትል አስፈላጊ ነው ብለው ያስባሉ? | 1. አዎ 2. የለም |  |
| 306 | ችግርባያጋጥምምድህረወሊድክትትልጠቃሚነውብለውያስባሉ? | 1. አዎ 2. የለም |  |

**ክፍል 4፡ የወንድ አጋር በድህረ-ወሊድ ጊዜ ስለሚከሰቱ አደገኛ ምልክቶች ያላቸውን እውቀት የሚመለከት ጥያቄ**

| 401 | በድህረ-ወሊድ ጊዜ አዎ ከሆነ በእናትየዋ ላይ የሚከሰቱት አደገኛ ምልከቶች ምን ምን ናቸው? | 1.ከፍተኛ ሙቀት  2.ከባድ እራስ ምታት  3.ብዙ ደም መፍሰስ  4.ሽታ ያለው የማህፀን ፈሳሽ  5.የአይን ብዥብዥታ  6.ከፍተኛ የሆነ የድብርት ስሜት  7.የእግር ጡንቻ አካባቢ መቅላትና ማመም |  |
| --- | --- | --- | --- |
| 402 | በህጻኑ ላይ የሚከሰቱት አደገኛ ምልከቶች ምን ምን ናቸው? | 1.ከፍተኛ ሙቀት  2.በሙቀት ጊዜ መቀዝቅዝ  3.ያተነፋፈስ ችግር  4.ያለተለመደ የሰውነት እንቅስቃሴ  5.እንብርት አካባቢ መቅላት፣ መድማት፣መምገል 6. የአጠባብ ችግር  7.የእጅና የእግር መዳፍ ቢጫ መሆን |  |
| 403 | በድህረ ወሊድ ጊዜ ስለሚመጡ የጤና ችግሮች ምን ምን ያቃሉ? | 1.የደም ግፊት  2.የጡትና የማህፀን ኢንፌክሽን  3.ሰራቂያን  4.የደም መርጋት  5.የአእምሮ ህመም  6.የቁስል መበላሸት |  |

**ክፍል 5፡ የወንድ አጋር ስለድህረ-ወሊድ ክትትል ያላችዉን ዝንባሌ የሚመለከት ጥያቄ**

| 501 | ወንዶች የትዳር አጋራችው የድህረ-ወሊድ ክትትል ሲደርጉ አብሮ መሳተፍ ጊዜ እንደማጥፋት ይቆጠራል | 1.በጣም እስማማለሁ 2.እስማማለሁ  3.አስተያየት የለኝም 4.አልስማማም  5.በጣም አልስማማም |  |
| --- | --- | --- | --- |
| 502 | ወንዶች በድህረ ወሊድ ወቅት ለሚስታችው የስነ-ልቦና, የገንዘብና ስራ በመርዳት ድጋፍ ማድረግ አለባቸው | 1.በጣም እስማማለሁ 2.እስማማለሁ  3.አስተያየት የለኝም 4.አልስማማም  5.በጣም አልስማማም |  |
| 503 | ወንዶች በድህረ-ወሊደ ወቅት የስራ ሀላፊነታችውን መወጣት እንጅ ሚስቶቻችዉንና ልጆቻቸውን የመንከባከብ ሀላፊነት የለባቸውም | 1.በጣም እስማማለሁ 2.እስማማለሁ  3.አስተያየት የለኝም 4.አልስማማም  5.በጣም አልስማማም |  |
| 504 | የድህረ-ወሊድ ክትትል ማድረግ ለሴቶች የሚተው ጉዳይ ነው | 1.በጣም እስማማለሁ 2.እስማማለሁ  3.አስተያየት የለኝም 4.አልስማማም  5.በጣም አልስማማም |  |
| 505 | በድህረ-ወሊድ ወቅት ጤና ተቃም መሄድ ለእናትዬዋም ሆነ ለህፃኑ ጥቅም አለው | 1.በጣም እስማማለሁ 2.እስማማለሁ  3.አስተያየት የለኝም 4.አልስማማም  5.በጣም አልስማማም |  |
| 506 | ሌሎች ወንዶች በድህረ-ወሊድ ክትትል ተሳትፎ እንዲያደርጉ አበረታታለሁ | 1.በጣም እስማማለሁ 2.እስማማለሁ  3.አስተያየት የለኝም 4.አልስማማም  5.በጣም አልስማማም |  |
| 507 | እናቶች ከወለዱ በኃላ በስድስት ሳምንት ውስጥ የድህረ-ወሊድ ክትትል ማደረግ አለባቸው | 1.በጣም እስማማለሁ 2.እስማማለሁ  3.አስተያየት የለኝም 4.አልስማማም  5.በጣም አልስማማም |  |

**ክፍል 6፡ የባህል ተፅእኖ/ተግዳሮት በወንድ አጋር የድህረ-ወሊድ ክትትል ተሳትፎ ላይ**

| 601 | የድህረ-ወሊድ ክትትል ማድረግ የሴቶች ድርሻ ነው? | 1.አዎ 2. አይደለም 3.አላቅም |  |
| --- | --- | --- | --- |
| 602 | የድህረ-ወሊድ ክትትል ክሊኒክ ሴቶች ብቻ የሚሄዱበት ቦታ ነው? | 1.አዎ 2. አየደለም 3.አላቅም |  |
| 603 | በቤተሰባችሁ ውስጥ ሰለእናቶች ጤና ክትትል የመወያየት ባህል አለ? | 1.አለ 2. የለም |  |
| 604 | በአካባቢያችሁ ወንዶች በድህረ-ወሊድ ክትትል ተሳትፎ እንዳያደርጉ የሚደርግ ባህል አለ? | 1.አለ 2. የለም |  |

**ክፍል 7፡ ከጤና ተቋም ጋር የተገናኙ ጥያቄዎች**

| 703 | የድህረ-ወሊድ ክትትል አገልግሎት የሚያገኙበት ተቋም ርቀቱ ምን ያክል ጊዜ ይዎስዳል? | 1.30 ደቂቃ እና ከዛ በታች  2. ከ 30 ደቂቃ በላይ |  |
| --- | --- | --- | --- |
| 704 | የድህረ-ወሊድ ክትትል አገልግሎት ወደሚያገኙበት ተቋም ለመሄድ በፈለጉ ጊዜ እንደልብ ትራንስፖረት ያግገኛሉ? | 1. አዎ 2. የለም 3.አላቅም |  |
| 705 | የጤና ባለሙያዎች ወንዶች ከባለቤታቸው ጋር ለድህረ-ወሊድ ክትትል ሲሄዱ ጥሩ አቀባበል አላችው? | 1. አላቸው 2. የላቸውም 3.አላቅም |  |
| 706 | በድህረ-ወሊድ ክትትል ክፍል የሚሰሩ ባለሙያዎች ከባለሙያ እና ከታካሚ ውጭ ሌላ ሰው እንዳይገባ ያደርጋሉ? | 1. አዎ 2. የለም 3.አላቅም |  |
| 708 | ባካባቢዎ ባለዉ የድህረ-ወሊድ ክትትል መስጫ ጤና ተቋም ለመስተናገድ ያለው የጊዜ ቆይታ ምን ያክል ነው? | 1. ከአንድ ሰዓት ያነሰ 2. ከአንድ ሰዓት በላይ 3.አላቅም |  |

**ክፍል 8፡ የወንድ አጋር ተሳትፎ በድህረ ወሊድ ክትትል ጊዜ**

| 801 | ከባለቤትዎ ጋር ስለድህረ-ወሊድ ክትትል ጥቅም ተዎያይተው ያውቃሉ? | 1.አዎ 2. የለም |  |
| --- | --- | --- | --- |
| 802 | ከባለቤትዎ ጋር በድህረ-ዎሊድ አገልገሎት ላይ የጋራ የሆነ ውሳኔ ይወስናሉ | 1.አዎ 2. የለም |  |
| 803 | ከባለቤትዎ ጋር የድህረ-ዎሊድ ክትትል ለማግኘት ሂደው ያቃሉ? | 1.አዎ 2. አልሄድሁም |  |
| 804 | መልሱ አልሄድሁም ከሆነ ምክንያትዎ ምንድን ነው? | 1.ሥራስለሚበዛ 2.ድህረ ወሊድ ክትትል ሚስቴን ብቻ የሚያሳስባት ነገር ነው ብየ ስለማስብ 3.ድህረ ወሊድ ችግር ስለማያመጣ 4. ሃላፊነት አይሰማኝም 5. ሀፍረት ስለሚሰማኝ 6.ባለቤቴ አብሬ እንዲሄድ አትፈቅድም  7. ሌላ ካለ…… |  |
| 805 | ከባለቤትዎ ጋር በድህር-ወሊድ ጊዜ ስለድህረ-ወሊድ የእርግዝና መከላከያ ዘዴዎች ተዎያይተው ያውቃሉ? | 1. አዎ 2. የለም |  |
| 806 | ክባለቤትዎ ጋር በመሆን ስለድህረ-ወሊድ ክትትል እና በድረ-ወሊድ ጊዜ ስለሚከሰቱ አደገኛ ምልክቶች ከባለሙያ ጋር ተዎያይተው ያቃሉ? | 1. አዎ 2. የለም |  |
| 807 | ለባለቤትዎ የድህረ-ዎሊድ ክትትል እንዲያገኙ አካላዊ ድጋፍ አድርገውላቸው ያውቃሉ? | 1. አዎ 2. የለም |  |
| 808 | ለባለቤትዎ የድህረ-ዎሊድ ክትትል እንዲያገኙ ስነ-ልቦናዊ ድጋፍ አድርገውላቸው(አበረታተዋቸው) ያውቃሉ? | 1. አዎ 2. የለም |  |
| 809 | ለባለቤትዎ የድህረ-ዎሊድ ክትትል እንዲያገኙ የገንዘብ ድጋፍ አድርገውላቸው ያውቃሉ? | 1. አዎ 2. የለም |  |
| 810 | በድህረ-ወሊድ ክትትል ጊዜ የእርስዎ ድርሻ ምንድን ነው? | 1. የቤት ውስጥ ሥራዎችን መስራት  2. ልጆሰቹን በቤት ውስጥ መንከባከብ  3. ሌላካለ…. |  |
