# Supplementary material for: Male partner involvement in postnatal care service utilization and associated factors: A community-based cross-sectional study in Motta district, Northwest Ethiopia
Source: PLoS One. 2023 Jan 20;18(1):e0276477. doi: 10.1371/journal.pone.0276477 (PMC9858086; doi:10.1371/journal.pone.0276477)
Supplement: S1 File — (DOCX) [file pone.0276477.s002.docx]

**Principal component analysis with steps:**

**1^st^ step**-exploring and selecting variables: As a first step exploring of the variables was done by running descriptive analysis that is frequency of each variable for urban and rural separately then some basic cleaning of data was done to include variables in the index and a range of variables included in the analysis. A household which has missing values for any of the assets and variables that are capable of distinguishing relatively “wealthy” households and relatively “poor” ones (by using rule of thumb that is if a variable/asset is owned by more than 95% or less than 5% of the sample) were excluded in the wealth index construction. For example,

**2^nd^ step**: Before creating the wealth index, all variables were transformed into scale variables. Since most often asset ownership questions are categorical variables, they were recoded into binary variables (variables that takes only 0 and) and variables with more than 2 categories were transformed into bivariate variables (table-1)**.**

**Table-1: A table showing some examples of variables which shows recoding in to binary variables**

| **List of Variables** | **Richer** | **Poorer** |
| --- | --- | --- |
| Owner of the house | 1 = private | 0 = rent or kebele’s |
| Roof material | 1= iron or concrete | 0 = grass or plastic |
| Type of lighting | 1 = electricity or gas | 0 = wood |
| Source of water | 1 = piped into dwelling or borehole with pump or protected dug well | 0 = pond or unprotected well |
| Toilet facilities | 1 = flush or ventilated improved latrine | 0 = open pit or none (open field) |
| Has a radio | 1 = yes | 0 = no |
| Has a TV | 1 = yes | 0 = no |
| Has a refrigerator | 1 = yes | 0 = no |
| Has a mobile phone | 1 = yes | 0 = no |
| Has a bicycle | 1 = yes | 0 = no |
| Has a bajaj | 1 = yes | 0 = no |
| Has a hourse/mule | 1 = yes | 0 = no |
| Has ox/cow | 1= yes | 0= no |
| Has a solar | 1= yes | 0= no |
| Has goat/sheep | 1= yes | 0= no |

**3^rd^ step**: in this step, a Principal Component Analysis (PCA) was conducted to create the wealth index. In the first analysis six components with eigenvalues (variance) greater than one were extracted. According to “Kaiser’s rule” only those components with eigenvalues greater than one should be retained. Variables that had correlation coefficients score of less than 0.3 were excluded in the second analysis. Correlation coefficient (𝑟) must be 0.30 or greater since anything lower would suggest a really weak relationship between the variables. Therefore, variables that had weak relationship were excluded in the second factor analysis. The second factor analysis was performed with the remaining six variables. Based on the same rule “Kaiser’s rule”, two components with eigenvalues greater than one were extracted. But the study decided to retain the first component (Ox/cow) because it had greater eigenvalue which explains the largest proportion of the total variance and it is used as the wealth index to represent the household’s wealth.

The following tables are outputs in the second factor analysis step:

| **Correlation Matrix** | | | | | | |  |  |  |  |
| --- | --- | --- | --- | --- | --- | --- | --- | --- | --- | --- |
|  | | domestic_cat2 | | kitchen_cat2 | | radio_cat2 | | bedspong_cat2 | bed_cat2 | mobile_cat2 |
| Correlation | ox/cow_cat2 | 1.000 | | .484 | | .005 | | .367 | .358 | -.104 |
|  | kitchen_cat2 | .384 | | 1.000 | | .393 | | .228 | .211 | .046 |
|  | radio_cat2 | .005 | | .393 | | 1.000 | | .137 | .153 | .304 |
|  | bedspong_cat2 | .367 | | .228 | | .137 | | 1.000 | .917 | .153 |
|  | bed_cat2 | .058 | | .211 | | .153 | | .917 | 1.000 | .681 |
|  | mobile_cat2 | -.104 | | -.046 | | .304 | | .153 | .081 | 1.000 |
| **KMO and Bartlett's Test** | | | | | | |  |  |  |  |
| Kaiser-Meyer-Olkin Measure of Sampling Adequacy. | | | | | .715 | |  |  |  |  |
| Bartlett's Test of Sphericity | | | Approx. Chi-Square | | 929.676 | |  |  |  |  |
|  |  |  | Df | | 15 | |  |  |  |  |
|  |  |  | Sig. | | .000 | |  |  |  |  |

The Kaiser-Meyer-Olkin Measure of Sampling Adequacy varies between 0 and 1. The values that are closer to 1 are better. A value of **0.6** is a suggested minimum acceptable value. In our study, we have a value of 0.715.

| Total Variance Explained | | | | | | | | | |
| --- | --- | --- | --- | --- | --- | --- | --- | --- | --- |
| Component | Initial Eigenvalues | | | Extraction Sums of Squared Loadings | | | Rotation Sums of Squared Loadings | | |
|  | Total | % of Variance | Cumulative % | Total | % of Variance | Cumulative % | Total | % of Variance | Cumulative % |
| 1 | 2.118 | 35.295 | 35.295 | 2.118 | 35.295 | 35.295 | 1.931 | 32.187 | 32.187 |
| 2 | 1.337 | 22.280 | 57.575 | 1.337 | 22.280 | 57.575 | 1.313 | 21.880 | 54.068 |
| 3 | .902 | 18.359 | 75.934 |  |  |  |  |  |  |
| 4 | .702 | 11.706 | 87.641 |  |  |  |  |  |  |
| 5 | .663 | 11.043 | 98.684 |  |  |  |  |  |  |
| 6 | .079 | 1.316 | 100.000 |  |  |  |  |  |  |
| Extraction Method: Principal Component Analysis. | | | | | | | | | |


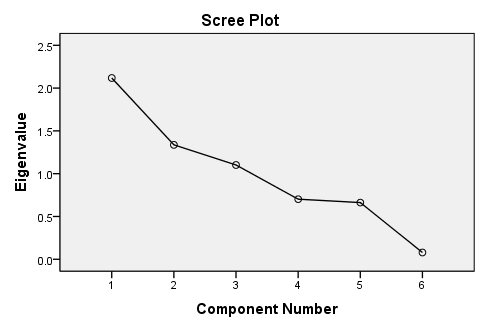


| Communalities | | |
| --- | --- | --- |
|  | Initial | Extraction |
| ox/cow_cat2 | 1.000 | .675 |
| kitchen_cat2 | 1.000 | .618 |
| radio_cat2 | 1.000 | .692 |
| bedspong_cat2 | 1.000 | .955 |
| bed_cat2 | 1.000 | .952 |
| mobile_cat2 | 1.000 | .664 |
|  | | |

We can also see the component matrix. This following table contains the component loadings, which are the correlations between the variable and the component. Possible values range from -1 to +1.

Component Matrix

|  | Component | |
| --- | --- | --- |
|  | 1 | 2 |
| bedspong_cat2 | .931 | .008 |
| bed_cat2 | .920 | -.014 |
| mobile_cat2 | .253 | .689 |
| ox/cow_cat2 | .190 | -.625 |
| kitchen_cat2 | .430 | -.496 |
| radio_cat2 | .345 | .474 |

Rotated Component Matrix

|  | Component | |
| --- | --- | --- |
|  | 1 | 2 |
| bed_cat2 | .970 | .061 |
| bedspong_cat2 | .968 | .098 |
| radio_cat2 | .059 | .816 |
| mobile_cat2 | .077 | .791 |
| ox/cow_cat2 | -.041 | -.071 |
| kitchen_cat2 | .204 | .052 |

Component Score Covariance Matrix

| Component | 1 | 2 |
| --- | --- | --- |
| 1 | 1.000 | .000 |
| 2 | .000 | 1.000 |

**4^th^ step**: Create wealth index quintiles

Before ranking the wealth index and create the quintiles, we did ‘weight cases’.

Then in order to better understand the wealth index, which was a continuous variable, we recoded the index into a categorical variable by ranking the WI (the first variable created from the PCA) into quintiles.

**RANK**

| **Created Variables^a^** | | | |
| --- | --- | --- | --- |
| Source Variable | Function | New Variable | Label |
| FAC1_1^b^ | Rank | RAN001 | Rank of FAC1_1 |
|  | Percentile Group^c^ | NTI001 | Percentile Group of FAC1_1 |
| a. Mean rank of tied values is used for ties. | | | |
| b. Ranks are in ascending order. | | | |
| c. 3 groups are generated. | | | |

**Step 5**: Select the final result and report the variables

**Frequencies**

| **Percentile Group of FAC1_1** | | | | | |
| --- | --- | --- | --- | --- | --- |
|  | | Frequency | Percent | Valid Percent | Cumulative Percent |
| Valid | poor | 215 | 36.1 | 36.1 | 36.1 |
|  | medium | 248 | 41.7 | 41.7 | 77.8 |
|  | rich | 132 | 22.2 | 22.2 | 100.0 |
|  |  | 595 | 100.0 | 100.0 |  |
| Total | |  |  |  |  |
