# Supplementary material for: Male partner involvement in postnatal care service utilization and associated factors: A community-based cross-sectional study in Motta district, Northwest Ethiopia
Source: PLoS One. 2023 Jan 20;18(1):e0276477. doi: 10.1371/journal.pone.0276477 (PMC9858086; doi:10.1371/journal.pone.0276477)
Supplement: Attachment — Submitted filename: Response to reviewers.docx [file pone.0276477.s004.docx]

Hi dear Editor and reviewers, we have carefully read and applied the suggestions from reviewers and comments from the editor as follow:

**Response to Editor**

- “We suggest you thoroughly copyedit your manuscript for language usage, spelling, and grammar”, we have tried to correct the language problems as much as possible
- “Can you please review all statistical reporting in your manuscript?” we have reviewed the statistical reporting and we have made some modifications
- “I request the authors do add the implications of this particular study at the end of the discussion section”, the implication and add value of this study is included at the end of the discussion section as you suggested.
- “In Abstract section, the authors state “favorable attitude [AOR=2.61, 95%CI= (1.55, 4.39)], short distance [(AOR=2.35, 95%CI=1.30, 4.26)]”, however, favorable attitude?? And short distance?? These phrases are incomplete that can potentially baffle the reader. Please, do them complete by stating the full variable (favorable attitude towards-------) this issue is also happened in operational definition section” we have tried to complete the incomplete phrases in the abstract and operational definition section
- “Please, see again the operational definition section, there even variables, which actually don’t need operationalization but did”, we have revised this section and tried to avoid variables which don’t need operationalization
- “Please, do erase recommendation and rewrite as conclusions” we have erased the word recommendation and merge the components with the conclusion section
- PNC service, Male involvement, Ethiopia: “Are these only keywords?”  we have added other keywords
- We have checked multicolinearity between independent variables using VIF which was less than 10
- The obtained information was kept anonymous and recorded in such a way that the respondent could never be known. “Please specify what type of consent you obtained”, we have specified the type of consent that we used
- “please include a copy, in both the original language and English, as Supporting Information” we have addressed this issue
- Data set is also included in supporting information

**Response to Reviewer #1**

- “In their Introduction an additional information regarding traditional gender roles expected of men and women in Ethiopia would benefit the paper in terms of measurements and hence outcomes /results”, we have tried to add some information regarding traditional gender roles in the introduction section but since we have no finding or associated factor related with this issue, this point does not included in the discussion section
- “Ethical considerations paragraph may be improved to include confidentiality and issues of anonymity”, we have included issue of confidentiality and anonymity
- “Write briefly about limitations and strength of their study approach/methodological considerations at the beginning of the Discussion section”, but we do not want to put the strength and limitation of the study at the beginning of the discussion section rather we have put it at the end of the discussion

**Response to Reviewer #2**

- “The language shows that the authors are not native speakers nor very experienced in academic write up in English”, we have tried to correct the language problems as much as possible
- “I miss a better cultural and social description on gender issues in pregnancy in Ethiopia. I miss a more thorough explanation of the state of the art in Postpartum care in general.”, we have tried to state cultural and social description of gender issue on postpartum care
- “A numeric comparison between countries make not so much sense”, we have deleted the numeric comparison between countries
- “Do not include too much detail in the abstract such as statistical method, confidence intervals or likewise”, we have avoided unnecessary details in the abstract section that you have mentioned
- “I do not really see how male participation in general is linked to better mother and child survival; this has to be explained (better access to resources, faster transport? Better negotiating power?)”, we have addressed this point by describing the link between male partner participation and better mother and child survival
- “The statement “reviewing different literatures” needs references, which studies did you learn from?”, we have put the references that we used for preparing the tool
- “No need to repeat table results in text. Shorten the presentation of results a little”, we have tried to shorten the presentation of results and avoided some repetitions
- “Please refrain from using too many decimals in estimations. One decimal is often enough”, we have tried to decrease decimals

**Thanks for your constructive suggestions and comments**
