# Supplementary material for: Male partner involvement in postnatal care service utilization and associated factors: A community-based cross-sectional study in Motta district, Northwest Ethiopia
Source: PLoS One. 2023 Jan 20;18(1):e0276477. doi: 10.1371/journal.pone.0276477 (PMC9858086; doi:10.1371/journal.pone.0276477)
Supplement: Attachment — Submitted filename: Response to reviewers.docx [file pone.0276477.s005.docx]

Hello dear Editor and reviewer, we have carefully read and applied the suggestions from you as follows:

**Response to Editor**

- “Two objectives (1 and 2) are mentioned on p.5-6, but it is not disclosed what these objectives are. Probably it should have been mentioned at the end of the introduction, that is too succinct regarding the goals of this particular study”
- *Since it has been stated at the end of the introduction section on page-5(‘therefore, the current study aimed to assess male partner involvement in PNC service utilization and associated factors in Motta district, North West Ethiopia’*), *we said ‘objective one and two’. But after reading your comment, we did some modifications.*
- **Male partner involved in PNC service:** those who had scored median value and above “(this would imply that, by definition, 50% are involved)”. Then: The overall magnitude of male involvement in PNC service utilization was 124 (20.8%). “Is the same name being used for two different things? Please clarify”.
- *Dear editor if we understand your question, it mean that 124(20.8%) of male partners scored ≥50% (median) from the given activities which measure the involvement of male partners.*
- “You should also provide some description of the scale of involvement. Table 4 provides the item by item, but there is no information on the distribution of the scale of the 10-point index”.
- *We think that this information is addressed in the operational definition section that is ‘Each of ten points was given a score of (1) when the participant performed the activity and (0) when the activity was not performed. A total score was computed for each participant and median score (50%) was used as a cut-off point to categorize into involved (those scored≥ 50% from the given activities) or not involved (<50% from the given activities). Since the data shows skewed distribution, median value was used as a cut-off point’.*
- “A more important comment is the lack of justification for the inclusion of knowledge of PNC service, Knowledge of danger signs during PNC, attitude to the service as independent variables. Note that the data is compiled AFTER the childbirth….. because of low involvement the opportunity was lost to get to know PNC services during pregnancy”.
- *Dear editor if we understand your question, we know that knowledge and attitude towards PNC service are the possible factors that affect male partner involvement in maternal health service as showed by different studies. Since those male partners with good knowledge may understand well the possible birth complications; so that they encourage their spouses to attend PNC service. As well as husbands’ positive perception on benefit of maternity care might lead to higher level of male partners’ participation. Generally this finding point to the important roles of male partner’s knowledge and attitude in influencing their involvement in postnatal care. Regarding to their opportunity to get knowledge and develop attitude towards PNC service, it could be through mass media, books, magazines or health education by Health extension workers, health care providers or another bodies. In the other way, this could be also the limitation of cross sectional study design to show cause and effect relationship.*
- “Note also, that the inclusion probably is also making the socioeconomic variables less interpretable: eg: education: It would be involvement of partner with different education level for a given level of knowledge of PNC. You’d expect part of the effect of education is through better knowledge…..”
- *If we understood your comment, even if there is confounding, we can filter out such like issues with double analysis or multiple regression analysis.*
- *And based on our understanding those who were educated might not have good knowledge and also those who were uneducated might not have poor knowledge.*
- “I am not so sure about the other service variables such as waiting time, privacy, and welcoming”.
- *We have removed these variables from the analysis based on your valuable comment.*
- “On a related topic: there is no talk about missing values and the “don’t know” responses. How were they treated?”
- *The ‘don’t know’ responses were recoded with ‘No’ responses for analysis and missing values were dropped*
- *But we have already removed those variables (like waiting time, privacy, welcoming, etc) with missing value and don’t know responses*
- “Please also provide in the appendix the details regarding the PC analysis for constructing the wealth index and the final index used”.
- *The details of PCA description has found in the appendix*

Response to Reviewer

- “The Authors need to write the limitations/methodological considerations of this study given that one of the main factors for male participation in maternal health services with their partners /wives are the underlining social cultural gender norms…..”
- *Dear reviewer we have accepted your comment and put as a limitation of this study. In addition to this we have recommended for further qualitative studies.*
- *And we would like to say thank you for the reference that you suggested.*
