# Supplementary material for: Male partner involvement in postnatal care service utilization and associated factors: A community-based cross-sectional study in Motta district, Northwest Ethiopia
Source: PLoS One. 2023 Jan 20;18(1):e0276477. doi: 10.1371/journal.pone.0276477 (PMC9858086; doi:10.1371/journal.pone.0276477)
Supplement: Attachment — Submitted filename: Response to Reviewer.rtf [file pone.0276477.s006.rtf]

Hello dear Editor, we have carefully read and applied the suggestions from you as follows:
•	“Regarding a possible conflict in the use of the term “median”: Median is a technical term referring to the location of the 50% percentile of a variable. Do you mean those that scored at least half of the total score? Then say so”.
-	Yes, It means that those scored half and above from the total score.
•	You again refer to “median score (50%)” Replace all such instances accordingly. It is wrong. All the instances of the word “median” in your reply are wrong! 
-	Thank you for your suggestion; we have replaced all such instances based on your recommendation. Page number8- 9, line number 185-189.
•	“A more important comment is the lack of justification for the inclusion of knowledge of PNC service, Knowledge of danger signs during PNC, attitude to the service as independent variables. Note that the data is compiled AFTER the childbirth….. because of low involvement the opportunity was lost to get to know PNC services during pregnancy”. “You have to state as one of the limitations that this variables are measured at the interview time, not prior to childbirth, and as a result can be affected by issues of reverse causality. You should raised these concerns in the section on the limitations of the study”.
-	We have understood your concern and have accepted your suggestion to correct it in such a way. So that we have addressed this issue in the limitation part. Page number 19, line number 403-406.
•	“It would be important to state what proportion of the observations had missing data and have been dropped”.
-	Based on our finding those who accompanied their partner for PNC visit were 18.7% (Page number 12, line 283). Since the remaining 81.3% of the participants didn't accompany their partner, they couldn't answer these questions like waiting time, privacy and welcoming which mean 81.3% of observations had missing data so that these variables (waiting time, privacy, welcoming, etc) have been dropped from the regression analysis.


# Reviewer
•	But we did not receive additional points raised by the reviewer.
