# Supplementary material for: Male partner involvement in postnatal care service utilization and associated factors: A community-based cross-sectional study in Motta district, Northwest Ethiopia
Source: PLoS One. 2023 Jan 20;18(1):e0276477. doi: 10.1371/journal.pone.0276477 (PMC9858086; doi:10.1371/journal.pone.0276477)
Supplement: Attachment — Submitted filename: Response to Reviewers.rtf [file pone.0276477.s007.rtf]

Hello, dear Editor, we have carefully read and applied the comments from you as follows:
•	“Please note that there are still many language errors in your submission”.
o	Thank you for your comment, and we have tried to use all efforts to edit the language errors.
•	“If you would like to make changes to your financial disclosure, please include your updated statement in your cover letter”.
o	We have not changed our financial disclosure.
Journal Requirements:
•	“If you have cited papers that have been retracted, please include the rationale for doing so…”
o	We have not used retracted papers.
•	“Any changes to the reference list should be mentioned in the rebuttal letter that accompanies your revised manuscript”.
o	We have not changed our reference lists.
